# Association of dysphagia and loneliness and their interaction with sleep quality among older adults in nursing homes: A cross-sectional study

**DOI:** 10.1371/journal.pone.0311024

**Published:** 2024-09-26

**Authors:** Bihan Wen, Yao Li, Mengyao Zhang, Huilan Xu

**Affiliations:** Department of Social Medicine and Health Management, Xiangya School of Public Health, Central South University, Changsha, Hunan, China; Stamford Health System: Stamford Hospital, UNITED STATES OF AMERICA

## Abstract

**Objective:**

Poor sleep quality is a risk factor for many adverse health outcomes and has become a widespread and serious public health problem, especially among older adults. This study aimed to explore the association between dysphagia, loneliness, and their interaction with sleep quality among older Chinese adults living in nursing homes.

**Methods:**

This cross-sectional study used multistage cluster random sampling to select 56 nursing homes in Hunan Province, China. Data on sociodemographic characteristics, health-related status, lifestyle, and behavioral and social psychological factors were collected. The Pittsburgh Sleep Quality Index was used to evaluate sleep quality. The 30 mL Water Swallowing Test and Eating Assessment Tool-10 items were used to screen for dysphagia, and the 14th item of the Center for Epidemiologic Studies Depression Scale was used to measure loneliness. Binary logistic regression models were used to analyze the relationship between poor sleep quality, dysphagia, and loneliness. The interaction between these variables was evaluated using multiplicative and additive interaction models.

**Results:**

This study included 3,356 older adults aged 60 and above. The mean Pittsburgh Sleep Quality Index score was 6.31 ± 3.11, and the incidence of poor sleep quality was 30.8%. A total of 642 (19.1%) older adults had dysphagia, and 1,358 (40.5%) experienced loneliness. After adjusting for all covariates, dysphagia and loneliness were associated with an increased risk of poor sleep quality. The interaction analysis demonstrated that the risk of poor sleep quality among older adults with dysphagia and loneliness was 3.476 times higher than that in those without dysphagia and loneliness. Dysphagia and loneliness had an additive interaction effect on poor sleep quality in older adults living in nursing homes.

**Conclusions:**

Poor sleep quality can be effectively prevented by focusing on older adults in nursing homes experiencing dysphagia, loneliness, or both and implementing targeted health interventions.

## Introduction

Population aging is an increasingly serious problem worldwide with the rapid development of modern society. As aging progresses, the sleep structure of older adults undergoes considerable changes, which can lead to sleep disorders. Sleep quality is an important indicator for evaluating the presence of sleep disorders. Globally, the incidence of poor sleep quality in older adults is high. A meta-analysis reported that the prevalence of sleep disorders among 104,046 older adults from different countries living in communities is 30.5% (95% confidence interval [CI]: 25.4–35.6%) [1]. Previous studies have found that older adults living in nursing homes experience more severe sleep disorders than those living in the community [2,3]. In addition, insufficient sleep leads to an increased prevalence of cardiovascular diseases, diabetes, obesity, and other diseases, which have become a widespread and serious social and public health problem.

Dysphagia, a swallowing disorder, is a common health problem among older adults and refers to the feeling of obstruction and stagnation caused by the obstruction of food transportation from the mouth to the esophagus and stomach [4]. A systematic review published in the United States showed that the incidence of dysphagia among older adults living in the community is between 5% and 72%, and the estimated average prevalence of dysphagia is 15% [5]. The prevalence of dysphagia among older adults living in nursing homes ranges from 15% to 70%, and this proportion has increased in recent years [6]. Dysphagia has several severe consequences. Accidental ingestion of food or liquids into the airway is one of the most common complications of dysphagia and can lead to suffocation and death in extreme cases [7]. Older adults with dysphagia are more likely to report symptoms such as difficulty falling asleep and maintaining sleep, daytime fatigue, and frailty [8,9].

Loneliness is a one-dimensional emotional response that arises when there is a discrepancy between the amount of desired social connection and the amount actually received. As people age, losing loved ones makes them more susceptible to loneliness. Approximately 40% of older adults aged 65 years and older reported feeling lonely [10]. The prevalence of moderate and severe loneliness is high in nursing homes. A recent meta-analysis estimated that the average prevalence of moderate and severe loneliness among older adults living in nursing homes was 61% and 35%, respectively [11]. Furthermore, long-term untreated loneliness can result in unrelieved psychological stress, which can have several adverse health consequences, including poor sleep, malnutrition, Alzheimer’s disease, cardiovascular disease, frailty, and impaired cognitive function [12].

Past research suggests that loneliness is associated with dysphagia and poor sleep quality. However, whether this can be inferred as a predictive variable of poor sleep quality remains unknown. Therefore, this study aimed to explore the interactions between dysphagia, loneliness, and poor sleep quality. It is assumed that the comorbidities of dysphagia and loneliness synergistically increase the risk of poor sleep quality among older adults in nursing homes. This investigation may provide new insights into the multidisciplinary care of poor sleep quality among older adults with dysphagia or loneliness, or both in nursing homes.

## Materials and methods

### Study population

This cross-sectional study was conducted in Hunan Province, China. A representative sample was selected using multistage stratified cluster sampling. The Hunan Province comprises 14 cities divided into two levels, based on urban and rural areas. In the first stage, two districts and two counties were randomly selected from each city, resulting in 28 districts and 28 counties. In the second stage, one street from each district and one township from each county were selected randomly, resulting in 28 streets and 28 townships. In the third stage, one nursing home was randomly selected from each street and township. Ultimately, 56 older adult nursing homes were selected. This study included older adults living in selected nursing homes who met the following inclusion criteria: (1) aged ≥ 60 years, (2) residence duration ≥ 3 months, and (3) signed informed consent and voluntary participation. However, older adults who met any of the following criteria were excluded: (1) individuals with mood or mental disorders, (2) individuals who were unconscious, (3) individuals with severe visual, hearing, or speech impairments, and (4) individuals with dementia or severe cognitive deficits diagnosed by clinicians (Mini-Mental State Examination < 18 points) [13,14].

The study was conducted between July 2021 and December 2021. Of the 6,595 older adults in 56 nursing homes, 2,669 were excluded because they met the exclusion criteria. Among the remaining 3,926 older adults who met the inclusion criteria, 570 were excluded because of incomplete data. Finally, the survey results of 3,356 participants were used for data analysis.

### Data collection

Data collection was conducted under the auspices of the Department of Civil Affairs of Hunan Province, the local Civil Affairs Bureau, and the administrators of each nursing home. The investigators were master’s degree students majoring in stomatology who were tasked with collecting information on oral hygiene. All investigators underwent rigorous training and assessment by practicing physicians before screening for dysphagia. Following their training, the investigators utilized a structured questionnaire to gather data via face-to-face interviews, each lasting between 30 minutes and 1 hour. The collected data included sociodemographic characteristics, health-related status, and lifestyle behavioral and social psychological factors.

### Measurements

#### Sleep quality assessment

The Pittsburgh Sleep Quality Index (PSQI) was used to assess sleep quality in older adults in nursing homes in the past month. The PSQI was compiled by Buysse and translated into Chinese by Liu [15,16]. The Chinese version has been widely used, with high reliability and validity in the older population (Cronbach’s α coefficient of 0.842). The scale includes 18 items and consists of 7 components: subjective sleep quality, sleep latency, sleep duration, sleep efficiency, sleep disturbances, use of sleeping medication, and daytime dysfunction. Each dimension is scored on a scale of 0 to 3 points, and the total PSQI score is obtained by adding the scores of each dimension, with a total score ranging from 0 to 21 points. A higher total PSQI score indicates poorer sleep quality. Participants were defined as having poor sleep quality when their PSQI scores were > 7 points.

#### Dysphagia assessment

We used the 30 mL Water Swallowing Test (WST) and the Eating Assessment Tool-10 (EAT-10) to assess dysphagia in older adults living in nursing homes. According to the data, the sensitivity and specificity of the screening tests increased when the EAT-10 and WST were used in tandem [17].

Kubota developed the WST to measure the degree of dysphagia by having an individual drink 30 mL of water [18,19]. Participants in this investigation were instructed to sit upright and drink 2–3 teaspoons of water at a time. If there were no problems, participants were asked to drink 30 mL of water at once. We then measured the duration and number of swallows, the time for one swallow (s), and the amount of water in one swallow (mL). The swallowing function was divided into five levels. Level I: can swallow the water smoothly; Level II: pauses more than two times to swallow, but without choking or coughing; Level III: one swallow, but there is choking and coughing; Level IV: it takes two or more attempts to swallow, and there is choking and coughing; Level V: choking and coughing frequently, and cannot swallow. Level I (finishing within 5 s) is normal, Level II or Level I (drinking water for more than 5 s) is suspicious, and Level III to V are abnormal. Suspicious or abnormal results indicated a positive screening test result for dysphagia. Previous studies have shown that the WST has good sensitivity but low specificity for screening dysphagia among older adults in nursing homes. The sensitivity, specificity, and positive and negative predictive values were 97.5%, 20.0%, 90.70%, and 50.00%, respectively [20,21].

The EAT-10 is a screening instrument for dysphagia developed by Belafsky et al. [22]. The EAT-10 consists of 10 items and its main components include dysphagia symptoms, clinical characteristics, psychological feelings, and social influence. Each item is scored on a scale of 0 to 4 points, and the total score can be obtained by adding the scores of the 10 items, with the total score ranging from 0 to 40 points. When the total score of the EAT-10 is ≥ 3 points, it indicates a positive result for dysphagia [23]. The EAT-10 helps identify signs of aspiration, silent aspiration, and dysphagia and has high reliability and validity in screening for dysphagia [24].

In this study, older adults in nursing homes who met one of the following conditions were considered to have dysphagia: (1) a positive 30 mL WST result; (2) an EAT-10 total score of ≥ 3 points.

#### Loneliness assessment

Loneliness was assessed utilizing the 14th item of the Center for Epidemiologic Studies Depression Scale [25]. The frequency of feeling lonely during the past week was measured and described as “I feel lonely.” The score range is 0 to 3 points, with “0 = Rarely or none of the time (< 1 day)”; “1 = Some or a little of the time (1–2 days)”; “2 = Occasional or moderate amount of time (3–4 days)”; “3 = Most or all of the time (5–7 days). Loneliness was categorized into two groups: 0 was designated as no loneliness, whereas scores ≥ 1 were defined as loneliness. Currently, using a single item to assess loneliness has gained widespread adoption [26,27].

#### Covariates

The covariates encompassed general information about the participants, including age, sex, educational level, marital status, monthly income, length of residence, number of descendants, and frequency of family visits. Health-related status was also investigated, including chronic disease history, fall history, physical frailty, sarcopenia, oral health status, and activities of daily living status. Additionally, lifestyle behavioral factors were considered, including smoking habits, alcohol consumption, physical activity levels, and sleep quality. Lastly, social psychological factors were evaluated, comprising social support, depressive symptoms, anxiety symptoms, and loneliness.

Smoking was defined as consuming at least one cigarette per day over the past year, whereas drinking was defined as engaging in at least one drinking episode per month in the preceding year. Frailty in older adults was assessed using the Tilburg Frailty Indicator, a Chinese-adapted version developed by Xi [28]. Sarcopenia was evaluated using the Simple Five-item Scoring Scale for Sarcopenia. The oral health status of the participants was assessed using the Oral Health Assessment Tool. The Barthel Index was used to evaluate the activities of daily living among older adults. The Chinese version of the International Physical Activity Questionnaire-short version was employed to assess the physical activity levels of older adults over the past week. The social support status of older adults was evaluated using the Social Support Revalued Scale, compiled by Xiao [29] in 1986, based on relevant foreign data and in accordance with actual conditions in China. The Geriatric Depression Scale-15 items was used to assess depressive symptoms in older adults. The Generalized Anxiety Disorder Scale-7 items was employed to evaluate anxiety symptoms among older adults.

### Statistical analysis

Continuous variables were described using mean ± standard deviation, or continuous variables were converted into ordinal variables for data analysis; categorical variables were described using relative measures. The chi-square test was used to compare groups. Multivariate logistic regression analysis was conducted to examine the effects of dysphagia and loneliness on sleep quality among older adults in nursing homes. The interaction between dysphagia and loneliness on poor sleep quality was analyzed using multiplicative and additive effects models. Prior to interaction analysis, stratification based on loneliness was performed along with a homogeneity test to assess the heterogeneity of the stratified data. Interaction analysis was conducted only once, and it was determined that loneliness was an effect-modifying factor of dysphagia on poor sleep quality.

The multiplicative effects model employed the evaluation method proposed by Rothman [30], integrating the product of dysphagia, loneliness, and their interaction term into a multivariate logistic regression model for verification. For the additive effects model, the Delta method was used to construct the interaction term “dysphagia + loneliness.” Subsequently, the covariance matrix was inputted into the specialized calculation table in Microsoft Excel compiled by Anderson et al. [31]. The point estimations and their 95% CI for three additive interaction evaluation indices were obtained: the relative excess risk of interaction (RERI), the attributable proportions of interaction (API), and the synergy index (S).

Evaluation indices were used to ascertain the presence of additive interactions. The absence of an additive interaction between the two factors was indicated when the point estimations and 95% CI of the RERI or the API encompassed 0 or when the same measures for the S encompassed 1. All statistical tests were performed using a two-tailed approach, with p-values of < 0.05 indicating statistical significance. All statistical analyses were conducted using SPSS version 26.0.

## Results

### Study sample characteristics

Fixty-six nursing homes in Hunan Province, China, were selected, and 3,926 older adults living in nursing homes met the inclusion and exclusion criteria. A total of 3,356 valid questionnaires were collected, with an effective response rate of 85.48%. The average age of the participants was 77.52 ± 9.16 years. Approximately 65% of the participants had not completed junior high school. Over half of the participants had a monthly income level below 3,000 Renminbi. Of the participants, 11.3% had no children or descendants, whereas 75% received monthly visits from relatives once or more. Of the participants, 62% had at least one chronic disease, 17.4% had a history of smoking, and 22.8% had a history of alcohol consumption. Symptoms of depression were exhibited in 31.8% of the participants, whereas 34.0% showed symptoms of anxiety.

In this study, 1,035 participants (30.8%) had poor sleep quality, with an average PQSI score of 6.31 ± 3.11. Among those with poor sleep quality, 40.9% reported subjective dissatisfaction, 52.5% reported taking more than 30 minutes to fall asleep, 46.3% reported sleeping ≤ 6 hours, 60.3% reported low sleep efficiency, 24.7% reported sleep disorders, 9.9% took hypnotic drugs more than once weekly, and 30.2% reported daytime dysfunction. Furthermore, 642 participants (19.1%) with dysphagia and 1,358 (40.5%) experiencing loneliness were screened (Table 1). Among 355 participants with dysphagia and loneliness, the detection rate of poor sleep quality was 56.1%. Among 1,003 participants without dysphagia but who experienced loneliness, the detection rate of poor sleep quality was 31.8%. Among 287 participants with dysphagia but without loneliness, the detection rate of poor sleep quality was 39.7%. Among 1,711 participants without dysphagia or loneliness, the detection rate of poor sleep quality was 23.6% (Table 1).

**Table 1:** Characteristics of older individuals living in nursing homes.

Characteristic	Total (n = 3,356)	Sleep quality		*χ* ^2^	p-value
		Good (n = 2321)	Poor (n = 1035)		
Age (years)				41.546	< 0.001
60–69	773 (23.0)	590 (25.4)	183 (17.7)		
70–79	940 (28.0)	677 (29.2)	263 (25.4)		
80–89	1,643 (49.0)	1,054 (45.4)	589 (56.9)		
Sex				0.564	0.453
Male	1,683 (50.1)	1,174 (50.6)	509 (49.2)		
Female	1,673 (49.9)	1,147 (49.4)	526 (50.8)		
Education				31.942	< 0.001
Primary school and below	2,186 (65.1)	1,571 (67.7)	615 (59.4)		
Junior high school	564 (16.8)	387 (16.7)	177 (17.1)		
Senior high school and above	606 (18.1)	363 (15.6)	243 (23.5)		
Stable marital status	1,276 (38.0)	967 (41.7)	309 (29.9)	42.352	< 0.001
Monthly income (≤ 3000 RMB)	2,082 (62.0)	1,510 (65.1)	572 (55.3)	29.145	< 0.001
Length of residence (> 1 year)	1,949 (58.1)	1,373 (59.2)	576 (55.7)	3.608	0.057
No descendants	378 (11.3)	303 (13.1)	75 (7.2)	24.162	< 0.001
No visiting relatives	852 (25.4)	625 (26.9)	227 (21.9)	9.431	0.002
Smoking	585 (17.4)	380 (16.4)	205 (19.8)	5.866	0.015
Alcohol consumption	765 (22.8)	452 (19.5)	313 (30.2)	47.153	< 0.001
Normal ADL status	1,493 (44.5)	1,031 (44.4)	462 (44.6)	21.240	< 0.001
Low physical activity levels	2,290 (68.2)	1,525 (65.7)	765 (73.9)	33.447	< 0.001
History of chronic disease	2,083 (62.1)	1,403 (60.4)	680 (65.7)	8.388	0.004
Fall history	583 (17.4)	331 (14.3)	252 (24.3)	50.736	< 0.001
Physical frailty	1,453 (43.3)	887 (38.2)	566 (54.7)	79.087	< 0.001
Sarcopenia	1,520 (45.3)	897 (38.6)	623 (60.2)	134.109	< 0.001
Sedentary behavior (≥ 5 h/d)	2,120 (63.2)	1,428 (61.5)	692 (66.9)	8.756	0.003
Normal nutritional status	1,442 (43.0)	1,061 (45.7)	381 (36.8)	24.630	< 0.001
Normal oral health status	1,176 (35.0)	853 (36.8)	323 (31.2)	10.034	0.007
No napping duration	821 (24.5)	593 (25.5)	228 (22.0)	9.651	0.008
Low social support	1,824 (54.4)	1,245 (53.6)	579 (55.9)	1.528	0.216
Depression symptoms	1,068 (31.8)	638 (27.5)	430 (41.5)	65.199	< 0.001
Anxiety symptoms	1,141 (34.0)	641 (27.6)	500 (48.3)	136.576	< 0.001
Dysphagia	642 (19.1)	329 (14.2)	313 (30.2)	119.438	< 0.001
Loneliness	1,358 (40.5)	840 (36.2)	518 (50.0)	57.053	< 0.001
Poor subjective sleep quality	590 (17.6)	167 (7.2)	423 (40.9)	560.187	< 0.001
Increased sleep latency	788 (23.5)	245 (10.6)	543 (52.5)	699.696	< 0.001
Sleep duration ≤ 6 hours	711 (21.2)	232 (10.0)	479 (46.3)	564.397	< 0.001
Reduced sleep efficiency	1,169 (34.8)	545 (23.5)	624 (60.3)	427.241	< 0.001
Sleep disturbances	274 (8.2)	18 (0.8)	256 (24.7)	548.004	< 0.001
Use of sleep medication	113 (3.4)	11 (0.5)	102 (9.9)	193.608	< 0.001
Daytime dysfunction	370 (11.0)	57 (2.5)	313 (30.2)	563.364	< 0.001

Values are n (%).

ADL, activities of daily living; RMB, Renminbi.

Significant differences were observed between poor and good sleepers in terms of age, education level, marital status, monthly income, number of living children, frequency of family visits, smoking, alcohol consumption, physical activity levels, daily living ability, history of chronic disease, fall history, physical frailty, sarcopenia, sedentary behavior, nutritional status, oral health status, napping duration, social support, depressive symptoms, anxiety symptoms, dysphagia, and loneliness (all p < 0.05) (Table 1).

### Association between sleep quality and dysphagia and loneliness

The participants were categorized into two groups based on sleep quality. A binary logistic regression analysis revealed that in the crude model, individuals with dysphagia exhibited an elevated risk of poor sleep quality (odds ratio [OR] = 2.625, 95% CI: 2.200–3.132, p < 0.001), and those experiencing loneliness displayed a similar increased risk (OR = 1.767, 95% CI: 1.523–2.049, p < 0.001). After adjusting for other covariates, this significant relationship persisted, and individuals with dysphagia exhibited a 2.117-fold higher risk of poor sleep quality than those without dysphagia (adjusted OR [aOR] = 2.117, 95% CI: 1.806–2.481, p < 0.001). Similarly, individuals experiencing loneliness had a 1.228-fold higher risk of poor sleep quality than those not experiencing loneliness (aOR = 1.228, 95% CI: 1.065–1.416, p = 0.018) (Table 2).

**Table 2:** Association between sleep quality and dysphagia and loneliness among older individuals living in nursing homes.

	Crude model			Adjusted model *		
	OR	95% CI	p-value	aOR	95% CI	p-value
Sex						
Male	1			1		
Female	1.058	0.914–1.225	0.453	1.070	0.892–1.284	0.465
Age/years						
60–69	1			1		
70–79	1.252	1.007–1.558	0.044	1.103	0.902–1.348	0.425
80–89	1.802	1.484–2.188	< 0.001	1.301	1.072–1.580	0.026
Dysphagia	2.102	1.637–2.699	< 0.001	1.640	1.222–2.199	< 0.001
No	1			1		
Yes	2.625	2.200–3.132	< 0.001	2.117	1.806–2.481	< 0.001
Loneliness						
No	1			1		
Yes	1.767	1.523–2.049	< 0.001	1.228	1.065–1.416	0.018

*Adjusted for age, sex, education, marital status, monthly income, duration of admission, number of living children, smoking status, alcohol consumption status, nutrition, history of chronic disease, social support status, and activities of daily living status.

aOR, adjusted odds ratio; OR, odds ratio; CI, confidence interval.

The various dimensions of the PSQI were considered dependent variables, whereas dysphagia and loneliness served as independent variables. A binary logistic regression analysis indicated that dysphagia was significantly associated with poor subjective sleep quality, increased sleep latency, sleep duration ≤ 6 hours, reduced sleep efficiency, sleep disturbances, use of sleep medication, and daytime dysfunction (all p < 0.05). Furthermore, the binary logistic regression analysis revealed a significant association between loneliness and poor subjective sleep quality, increased sleep latency, sleep disturbances, use of sleep medications, and daytime dysfunction (all p < 0.05) (Table 3).

**Table 3:** Association between the dimensions of the PSQI scale and dysphagia and loneliness among older adults in nursing homes.

	Dysphagia			Loneliness		
	OR	95% CI	p-value	aOR	95% CI	p-value
Poor subjective sleep quality	1.978	1.592–2.458	< 0.001	2.558	2.116–3.093	< 0.001
Increased sleep latency	1.649	1.350–2.014	< 0.001	1.381	1.169–1.632	< 0.001
Sleep duration ≤ 6 hours	1.891	1.535–2.329	< 0.001	1.172	0.985–1.395	0.074
Reduced sleep efficiency	1.413	1.173–1.703	< 0.001	0.953	0.820–1.108	0.530
Sleep disturbances	2.082	1.544–2.807	< 0.001	2.332	1.778–3.059	< 0.001
Use of sleep medication	1.561	1.011–2.409	0.044	1.812	1.227–2.676	0.003
Daytime dysfunction	1.863	1.445–2.403	< 0.001	2.066	1.644–2.595	< 0.001

*Adjusted for age, sex, education, marital status, monthly income, duration of admission, number of living children, smoking status, alcohol consumption status, nutrition, history of chronic disease, social support status, and activities of daily living status.

PSQI, Pittsburgh Sleep Quality Index; aOR, adjusted odds ratio; OR, odds ratio; CI, confidence interval.

### Effect of synergistic interaction between dysphagia and loneliness on poor sleep quality

An analysis of the multiplicative interaction between dysphagia and loneliness on poor sleep quality among older adults residing in nursing homes was conducted using binary logistic regression. With the sleep quality of older adults as the dependent variable, dysphagia, loneliness, and their multiplicative interaction terms were considered independent variables. The analysis was also controlled for potential confounders using a multivariate logistic regression model. The multiplicative interaction between dysphagia and loneliness did not significantly affect sleep quality among older adults living in nursing homes (p > 0.05) (Table 4).

**Table 4:** Multiplicative interactive effect of loneliness and dysphagia on poor sleep quality in older adults based on binary logistic regression analysis.

	Model 1^a^		Model 2^b^	
	OR (95% CI)	p-value	aOR (95% CI) *	p-value
Dysphagia	2.139 (1.647–2.778)	< 0.001	1.987 (1.627–2.428)	< 0.001
Loneliness	1.514 (1.272–1.801)	< 0.001	1.289 (1.056–1.574)	0.013
Dysphagia * Loneliness	1.279 (0.892–1.834)	0.181	1.356 (0.927–1.984)	0.116

^a^: Unadjusted.

^b^: Adjusted for age, sex, education, marital status, monthly income, duration of admission, number of living children, smoking status, alcohol consumption status, nutrition, history of chronic disease, social support status, and activities of daily living status.

aOR, adjusted odds ratio; OR, odds ratio; CI, confidence interval.

**Fig 1:**
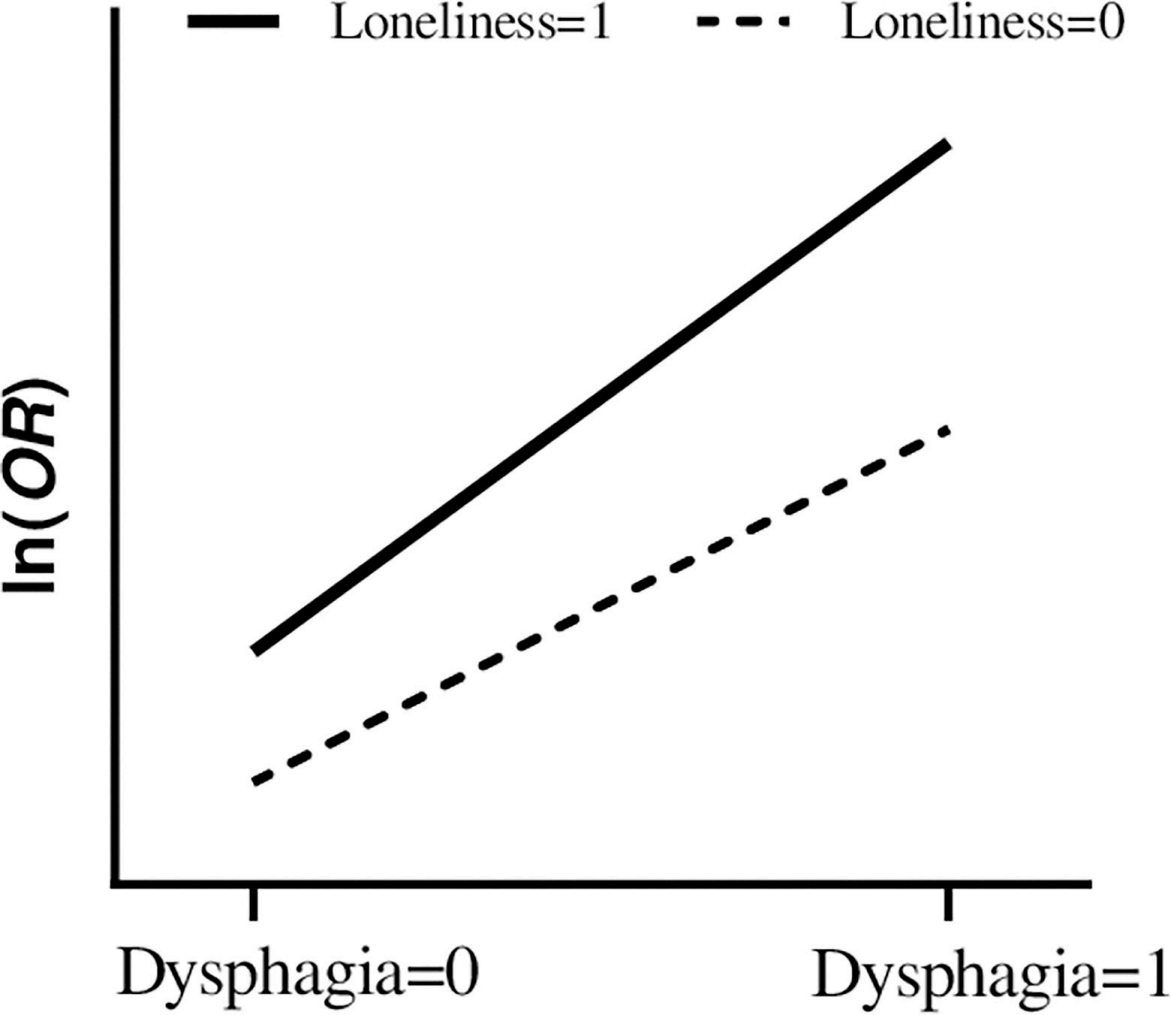
Average response plot for interactions. OR, odds ratio.

The additive interaction between dysphagia and loneliness on poor sleep quality among older adults in nursing homes was analyzed using binary logistic regression. After controlling for potential confounders, a multivariate logistic regression analysis was performed. Participants with dysphagia and loneliness exhibited a 3.476-fold increased risk of poor sleep quality compared with those without dysphagia and loneliness (Table 5). After adjusting for covariates, the calculated RERI = 1.199 (95% CI: 0.399–2.000), API = 0.345 (95% CI: 0.149–0.541), and S = 1.939 (95% CI: 1.162–3.236) suggest a positive additive interaction (synergism) between dysphagia and loneliness on the occurrence of poor sleep quality among older adults in nursing homes (Table 6). An average response plot was used to explain the direction of interaction. Dysphagia and loneliness exhibit additive interaction effects (Fig 1), where the distinct separation of the two lines signifies a super-additive (synergistic) effect on a logarithmic scale.

**Table 5:** Additive interactive effect of loneliness and dysphagia on sleep quality in older people based on binary logistic regression analysis.

Variable		Sleep quality		Crude OR (95% CI)	Adjusted OR (95% CI) *
		Good	Poor		
Dysphagia	Loneliness				
**-**	**-**	1,308	403	1	1
**+**	**-**	173	114	2.139 (1.647–2.778)	1.987 (1.627–2.428)
**-**	**+**	684	319	1.514 (1.272–1.801)	1.289 (1.056–1.574)
**+**	**+**	156	199	4.140 (3.265–5.250)	3.476 (2.858–4.229)

*Adjusted for age, sex, education, marital status, monthly personal income, duration of admission, number of living children, smoking status, alcohol consumption status, nutrition, history of chronic disease, social support status, and activities of daily living status.

CI, confidence interval; OR, odds ratio.

**Table 6:** Index of additive interaction between dysphagia and loneliness on poor sleep quality in older adults in nursing homes.

	Crude model	Adjusted model*
RERI	1.489 (0.571–2.406)	1.199 (0.399–2.000)
API	0.359 (0.176–0.543)	0.345 (0.149–0.541)
S	1.901 (1.223–2.954)	1.939 (1.162–3.236)

*Adjusted for age, sex, education, marital status, monthly income, duration of admission, number of living children, smoking status, alcohol consumption status, nutrition, history of chronic disease, social support status, and activities of daily living status.

RERI, relative excess risk of interaction; API, attributable proportions of interaction; S, synergy index.

## Discussion

This study examined the prevalence of poor sleep quality among older adults living in nursing homes in Hunan Province, China. Furthermore, the association between dysphagia and loneliness and their interaction with sleep quality was investigated. Based on this large-scale population survey, we found that poor sleep quality affects nearly one-third of older adults living in nursing homes in Hunan Province, China. A synergistic effect was observed between dysphagia and loneliness, which had a detrimental effect on sleep quality among older adults.

In this study, the prevalence of poor sleep quality among older adults in nursing homes was 30.8%, consistent with the findings of a recent review by Yan et al. [1]. This review included 48 studies, resulting in a 30.5% prevalence of poor sleep quality among older adults in community-based and primary care settings. The results of this study were slightly underreported compared with the findings of Fatma et al. (60.3%) [32] and Liu et al. (60.9%) [33], but surpassed those of Yue et al. (22.5%) [34], Dong et al. (27.7%) [35], and Wang et al. (27.8%) [36]. The variance in the estimated values of sleep quality may stem from the use of distinct measurement tools and the varying truncation values of these tools. In this study, participants were defined as having poor sleep quality if their PSQI scores were > 7 points. Additionally, these variations may be attributed to differing ages and chronic disease prevalence among the research participants, alongside variations in maintenance practices adopted by nursing homes. Some researchers believe that the altitude of a residential area can influence sleep quality in older adults. A cross-sectional study comparing sleep disorders in nursing homes in high- and low-altitude areas in China revealed that the prevalence of sleep disorders in high-altitude areas was 41.54%, whereas that in low-altitude areas was 18.76% [37].

The prevalence of loneliness among older adults in nursing homes in the present study was 40.5%, mirroring the results reported in previous investigations. Additionally, a Chinese study of over 7,000 older adults revealed that 38.28% of rural older adults frequently experienced loneliness [38]. The detection rate of dysphagia in the current study among older adults in nursing homes was 19.1%, which is slightly lower than that observed in other investigations. For instance, Park et al. evaluated 395 older adults residing in nursing homes in an urban area of South Korea using the Gugging Swallowing Screen test, which revealed a dysphagia prevalence of 52.7% [39]. Nogueira et al. assessed 272 older nursing home residents in Portugal using the Dysphagia Self-Test and found that 40% exhibited signs of dysphagia [40]. Our findings align with those of other studies, including Okabe et al., who determined the risk of dysphagia in 18.5% of 238 nursing home residents in Japan using a modified water swallowing test [41]. The primary reason for these discrepancies is the use of varying measurement tools. Domestically and internationally, the commonly employed screening instruments mainly consist of screening scales and tests. Consequently, differences in these tools lead to variations in measurement outcomes.

Sleep disorders among older adults are the result of several risk factors. Multifactorial logistic regression analysis, after controlling for confounding factors such as age and sex, showed that dysphagia and loneliness can influence sleep quality among older adults in nursing homes. Interventions for older adults who experience dysphagia and loneliness can lessen the health hazards of dysphagia and loneliness themselves, as well as reduce the onset and progression of poor sleep quality.

Older adults with dysphagia have a higher risk of poor sleep quality than those without dysphagia. Swallowing problems, such as salivation, hypersalivation, choking, and aspiration, can affect the sleep quality of people with dysphagia. In a cross-sectional study conducted in Taiwan, China, patients with dysphagia scored higher on the PSQI, indicating poorer sleep quality, than those without dysphagia [8]. Italian researchers found a strong relationship between excessive daytime sleepiness and the development of swallowing impairment in a 3-year cohort study. Further, they found that daytime sleepiness was generally more prolonged in patients with Parkinson’s disease and dysphagia than in those without dysphagia. Excessive daytime sleepiness, therefore, led to relatively shorter nighttime sleep duration and poorer quality sleep [42]. Dysphagia can also affect eating habits, such as slowing the rate of eating and swallowing. Moreover, patients diagnosed with dysphagia may experience malnutrition, which can also lead to sleepiness and reduced ability to work [43,44].

Older adults experiencing loneliness are more likely to develop sleep disorders. An increasing number of studies have revealed that a greater degree of loneliness predicts a gradual decline in sleep quality among older adults. Through a systematic review and meta-analysis, Deng et al. confirmed that loneliness significantly correlates with impaired sleep quality among older adults and that those experiencing loneliness experience poorer sleep quality than those without [45]. Individuals experiencing social isolation exhibit disrupted sleep patterns and increased vascular activation. Shankar et al. conducted a 4-year follow-up survey involving 5,698 older individuals and discovered a close correlation between loneliness and reduced, fragmented sleep. Participants in the high-level loneliness group exhibited a greater incidence of sleep issues during follow-up than those in the low-level loneliness group. Those experiencing loneliness struggled more with initiating sleep and frequently awoke feeling exhausted, in contrast to their non-lonely counterparts [46]. Several researchers have posited that perceived social isolation or loneliness serves as a stressor that triggers negative emotions, reactivity, and diminished self-esteem, ultimately leading to persistent elevations in the activation of the sympathetic nervous system, sympathetic adrenomedullary, and hypothalamic-pituitary-adrenocortical neuroendocrine axes [47]. Loneliness can impair the immune system functionality and exacerbate neuroendocrine dysregulation, which contributes substantially to the degradation of sleep quality [48].

The incidence of disease in the presence of multiple risk factors differs considerably from that estimated based solely on individual effects. In this study, we used multiplicative and additive models to assess the association of dysphagia, loneliness, and their interaction with sleep quality, and the results were analyzed statistically to explore the possibility of a biological interaction. The interaction analysis revealed a significant additive interaction between dysphagia and loneliness on poor sleep quality among older adults. Specifically, older adults in nursing homes with dysphagia and loneliness had a 3.476-fold increased risk of poor sleep quality than older adults without dysphagia and loneliness.

Dysphagia is strongly correlated with a decline in social and psychological functioning, rendering older adults with this condition more prone to social and psychological impairments. A European study of 360 nursing home residents with dysphagia revealed that only 45% perceived eating as an enjoyable activity, 41% experienced anxiety or panic during meals, and 36% avoided dining with others because of their dysphagia [44,49]. Older adults with dysphagia experience increased loneliness and loss of self-esteem, feel rejected by others, and lose the enjoyment of socializing and mealtimes, which affects the quality of the patient’s relationships with caregivers and family members. Individuals with dysphagia reported a sense of social isolation. Dysphagia can restrict the extent of socialization among older adults, leading to drastic changes in their daily behaviors and lifestyles. Fear of aspiration and coughing, coupled with the accompanying anxiety, can result in frustration and progressive social isolation among older adults [50]. Healthcare providers must be aware of the negative impact of dysphagia on self-esteem, socialization, and quality of life. The findings of this study indicate that providing care to older individuals with dysphagia and timely psychological counseling to targeted populations can reduce the risk of poor sleep quality. Appropriate oral hygiene practices, strategies to enhance eating habits, and initiatives to alleviate loneliness should be considered integral components of a comprehensive sleep management program tailored for those with poor sleep quality.

However, this study had several limitations. First, we briefly analyzed dysphagia, loneliness, and their interaction with poor sleep quality, suggesting a statistically additive interaction between dysphagia and loneliness. However, the underlying biological mechanisms require further investigation. Second, because this was a cross-sectional study, causal inferences cannot be made and are only suggestive. Third, this study used a self-reported questionnaire, and the results might have had some recall bias. Finally, our study sample was confined to older adults residing in nursing homes in Hunan Province, China, which restricts the generalizability of the findings.

## Conclusions

Dysphagia and loneliness were related to poor sleep quality among older adults in nursing homes in Hunan Province, China. Furthermore, the synergistic interaction between dysphagia and loneliness may increase the risk of poor sleep quality in this demographic. Healthcare professionals should assess the sleep quality status of older adults who have dysphagia or loneliness, or both. Timely interventions may effectively prevent poor sleep quality among older adults in nursing homes.
